# A simple method for in-house *Pfu* DNA polymerase purification for high-fidelity PCR amplification

**Published:** 2019-04

**Authors:** Prabu Siva Sankar, Marimuthu Citartan, Aminah Ahmed Siti, Boris V. Skryabin, Timofey S. Rozhdestvensky, Goot Heah Khor, Thean Hock Tang

**Affiliations:** 1 Advanced Medical & Dental Institute (AMDI), Universiti Sains Malaysia, Bertam, Kepala Batas, Penang, Malaysia; 2 Faculty of Medical (TRAM), University of Muenster, Muenster, Germany; 3 Centre of Preclinical Science Studies, Faculty of Dentistry, Universiti Teknologi MARA, Sungai Buluh Campus, Selangor, Malaysia

**Keywords:** Protein expression, *Pyrococcus furiosus*, DNA polymerase, Polymerase chain reaction

## Abstract

**Background and Objectives:**

*Pfu* DNA polymerase is an enzyme that exhibits the lowest error rate in the 3′ to 5′ exonuclease (proofreading) activity during DNA synthesis in Polymerase Chain Reactions (PCRs). This study was aimed to express and purify *Pfu* DNA polymerase in a bacterial expression system under a simple purification method.

**Materials and Methods:**

*Pfu* polymerase gene sequence, derived from *Pyrocuccus furiosus (Pfu)* genomic DNA, was cloned and overexpressed in *E. coli* BL21 (DE3) pLysS. Upon overexpression, bacterial lysate containing the *Pfu* DNA polymerase was heated at 94°C for 5 minutes. *Pfu* DNA polymerase having high thermal stability was retained while the other bacterial proteins were denatured. The resulting thermo stable *Pfu* DNA polymerase was separated from the other debris of the denatured proteins by simple centrifugation.

**Results:**

The enzymatic activity of the resulting *Pfu* DNA polymerase was estimated by comparing with the commercial *Pfu* DNA Polymerases. An estimated 50000 units of functional *Pfu* DNA polymerase was produced from a 400 ml culture.

**Conclusion:**

The in-house produced *Pfu* DNA Polymerase could be used for routine amplification that requires high-fidelity such as cloning and DNA sequencing.

## INTRODUCTION

DNA amplification using Polymerase Chain Reaction (PCR) has long emerged as one of the fundamental technologies of molecular biology. Generic PCR consists of three main thermal steps: 1) DNA template denaturation where the double stranded DNA is denatured into two single stranded DNA; 2) primer annealing where the primers anneal to the complementary sequences in the template strands 3) and elongation - DNA polymerase synthesizes new copies of DNA from the template (1). Commonly, *Taq* polymerase is used as the enzyme of choice in the PCR amplification. This polymerase has no proof-reading capacity (3′-5′ exonuclease), with an error rate of 8×10^−6^ mutations/bp/duplication (2). In applications that require high fidelity such as cloning, a polymerase capable of proof-reading is highly desirable and one such DNA polymerase is the DNA Polymerase from *Pyrococcus furiosus* (*Pfu*).

The *Pfu* DNA polymerase gene encodes a polypeptide of 775 amino acids with a predicted molecular weight of 90, 109 Da (3, 4). The polymerase possesses a 3′-5′ exonuclease activity that allows for removal of mis-incorporated bases (“proof-reading”) and has been reported to exhibit a low error rate in PCR (1.3×10^−6^ mutations/bp/duplication) which is approximately 8-fold lower as compared to *Taq* Polymerase (2, 5). Various researchers have highlight on the expression and purification strategy of the polymerase with a focus on the biophysical characteristic of the protein and/or recombinant DNA technology such as the immobilized metal affinity-tagged purification, weak cation exchange resins, polyethylenemine precipitation, ammonium sulfate precipitation and ion exchange chromatography (6–11). In this study, we reported an in-house expression and purification method of *Pyrococcus furiosus (Pfu)* DNA polymerase in *E. coli* BL21 (DE3) pLysS which capitalized on the thermal stability of the *Pfu* DNA polymerase as the key strategy for the purification which averts the usage of other tedious, specialized and expensive strategies. The overexpressed *Pfu* DNA polymerase containing lysate was subjected to heating which denatures the rest of the less thermally stable protein, forming debris which is subsequently removed. As a test of functionality, the protein was used to amplify a template plasmid.

The work aims to obtain purified *Pfu* DNA polymerase at a low cost using a simple in-house purification method that averts specialized techniques and equipment for research and teaching use.

## MATERIALS AND METHODS

### Plasmid.

*Pyrococcus furiosus* chromosomal DNA was kindly provided by Dr. Boris at the University of Muenster, Germany. *Pfu* DNA polymerase gene sequence was amplified and cloned into pSE420 plasmid (Invitrogen, MA, USA). The forward and reverse primers used were Fpfu 5′-AATATTGAATTccatggTTTTAGATGTGGATTAC-3′, which contains the *Nco*I Restriction site and Rpfu 5′-AATATTGAATTCactagtCTAGGATTTTTTAATGTTAAGC-3′ that contains the *Spe*I restriction site (restriction sites are represented with lower case and underlined). Following restriction digestion of the both, plasmid and the PCR product, ligation was carried out for 16 hours at 4°C. The ligation mixture was electro-transformed into *E. coli* DH5α and the recombinant plasmid named pSEPfu was confirmed with sequencing.

### Protein expression.

The pSEPfu plasmid was extracted and electro-transformed into *E. coli* BL21 (DE3) pLysS (Merck, MA, USA). A single positive colony was picked and inoculated into a 10 ml LB broth (containing 100 μg/ml ampicillin and 35 μg/ml chloramphenicol) and cultured for 16 hours at 37°C, 200 rpm. The starter culture was inoculated at a ratio of 1:100 to LB broth (containing 100 μg/ml ampicillin and 35 μg/ml chloramphenicol). The culture was incubated at 37°C and was shaken at 200 rpm. When the OD_600_ reached 0.5, induction with 0.5 mM IPTG was carried out for a total of 6 hours. One mL of culture was taken at each hour and subjected to protein profiling by 12% SDS PAGE analysis. The intensities of the bands that correspond to the expected size of the *Pfu* Polymerase were compared between the different hours of induction period. The band of expected size with the highest intensity constituted the most optimal hour of induction.

### Protein purification.

The culture was pelleted at 10,000 × g for 10 minutes and the cells were lysed with BugBuster® Protein Extraction Reagent (Merck, MA, USA) as per manufacturer protocol. The lysate was centrifuged at 15,000 × g for 15 minutes and the supernatant was heated at 94°C for 5 minutes. The insoluble fraction was separated from the soluble fraction by centrifugation at 10, 000 × g for 5 minutes at 4°C. The soluble fraction was dialyzed against a buffer containing 20 mM Tris-Cl pH 7.5, 100 mM KCl, 0.1 mM EDTA, 1 mM DTT and 50% glycerol (*Pfu* Buffer). Dialysis was repeated for 4 times, 8 hours each. The dialyzed fraction was subjected to protein purity analysis via 12% SDS PAGE.

### Functional assay of the *Pfu* Polymerase based on PCR amplification.

To assess the functionality of the purified *Pfu* Polymerase, PCR amplification was carried out using the template p600 plasmid (recombinant plasmid containing a 600 bp insert of ICR mouse *c-myc* gene). The PCR reactions carried out were in 20 μl volume containing 20 mM Tris-Cl pH 8.8, 10 mM KCl, 10 mM (NH_4_)_2_ SO_4_, 2 mM MgSO_4_, 0.1% Triton® X-100 and 0.1 mg/ml nuclease-free BSA, 0.2 mM dNTPs, 0.6 μM p600 primers, and 150 ng of the template. To estimate the enzymatic activity of the *Pfu* Polymerase, the band intensity of the PCR amplification was compared to that of the commercial *Pfu* Polymerases from Promega (3U/μl) (Promega, WI, USA) and Thermo Scientific Fisher (2.5U/μl) (Thermo Scientific, MA, USA). Different titration stocks of the *Pfu* Polymerase were made accordingly by dilution with the Pfu buffer (2×, 5×, 10×, 20×). The PCR parameters were: Initial denaturation: 94°C for 5 min, 35 cycles of denaturation: 94°C for 40 seconds, annealing: 68°C for 40 seconds, extension: 72°C for 40 seconds, and final extension: 72°C for 5 minutes. Band intensity was analyzed via ImageJ software using plot profile and 3D surface plot (12).

## RESULTS

### Optimum time for overexpression of *Pfu* polymerase.

**Fig. 1.**
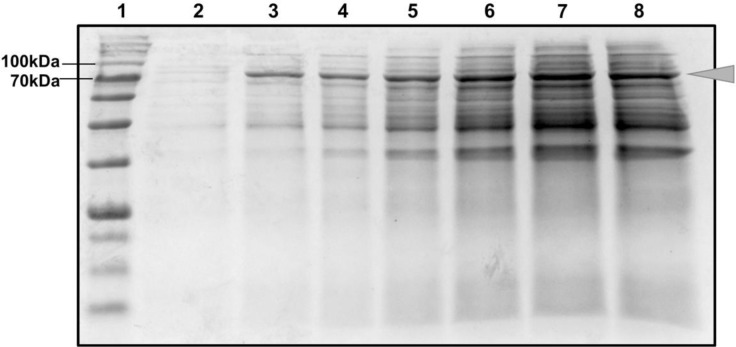
Time course induction of *Pfu* protein expression. SDS PAGE analysis to gain insight on the time course analysis of *Pfu* DNA Polymerase expression following IPTG induction after 1, 2, 3, 4, 5 & 6 hours. Lane 1: Protein size marker, Lane 2: 0 hour (before IPTG was added), Lane 3: 1 hour, Lane 4: 2 hours, Lane 5: 3 hours, Lane 6: 4 hours, Lane 7: 5 hours, Lane 8: 6 hours. *Pfu* Polymerase (arrow) band with the size of 90 kDa was seen to be expressed. 5 hours was chosen as the optimal induction period.

Overexpression of the *Pfu* DNA polymerase was carried out in *E. coli* BL21 (DE3) pLysS. The time course expression was performed by IPTG-based induction with sample collection at different time points up to 6 hrs. SDS-PAGE analysis was carried out with the collected samples to get an overview on the expression level of *Pfu* polymerase at the different induction periods (Fig. 1). A band of an expected size of 90 kDa was seen, which represent the overexpressed *Pfu* DNA polymerase across the period of 6 hours. The optimum time for overexpression was determined to be 5 hours as at this time point, the highest band intensity was reflected which corresponded to the highest level *Pfu* DNA Polymerase expression. Subsequently, to extract the overexpressed protein, we chemically lysed the cell using the BugBuster® Protein Extraction Reagent.

### Heat-based purification and centrifugation of *Pfu* DNA Polymerase.

**Fig. 2.**
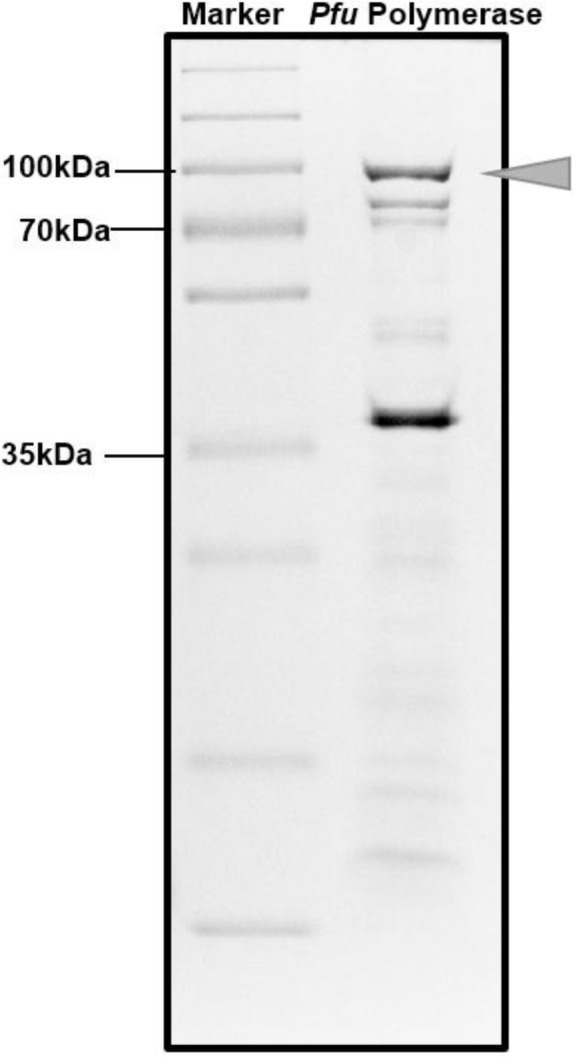
SDS PAGE of Purified *Pfu* Polymerase. Arrow shows the purified *Pfu* Polymerase after dialysis.

Following the separation of the soluble from the insoluble fraction by centrifugation at 10,000 × g for 5 min, the soluble proteins were heated at 94°C for 5 minutes. This heating step denatured most of the *E. coli* proteins while sustaining the thermostable *Pfu* polymerase (11). The lysate was centrifuged to obtain the soluble fraction. The soluble fraction, which comprises the purified protein was dialyzed extensively with the *Pfu* Buffer. SDS PAGE analysis of the dialyzed protein shows that presence of the band, which corresponds to the estimated size of the 90 kDa protein *Pfu* DNA Polymerase (Fig. 2).

### Titration of enzymatic activity.

**Fig. 3.**
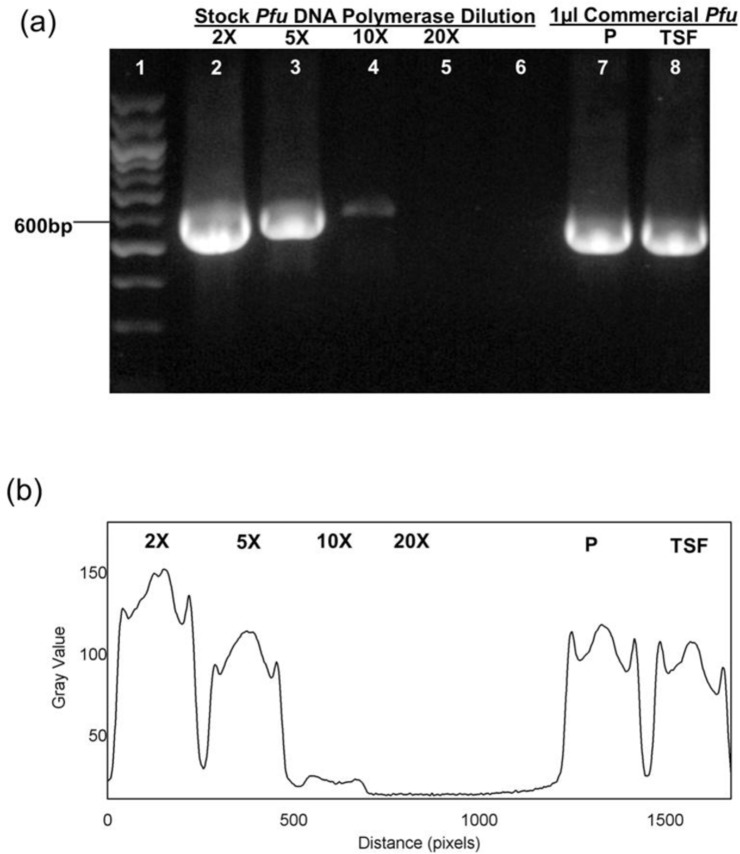
Estimation of enzymatic activity for purified *Pfu* DNA Polymerase. (a) Gel electrophoresis of PCR product with *Pfu* Polymerase titration with the relative intensity of the amplicons. *Pfu* Polymerase from the stock was diluted in *Pfu* buffer (2×, 5×, 10×, 20×) accordingly. The PCR amplification was run in parallel with 1 μL of *Pfu* DNA Polymerase from Promega (3U/μL) and 1 μL of *Pfu* DNA Polymerase from Thermo Scientific (2.5U/μL). Lane 1: your marker, Lane 2: 2× dilution, Lane 3: 5× dilution, Lane 4: 10× dilution, Lane 5: 10× dilution, Lane 6: Negative Control, Lane 7: Promega (P) *Pfu* Polymerase, Lane 8: Thermo Scientific (TSF) *Pfu* Polymerase. (b) Plot Profile analysis of the bands.

Titration stocks were made to assess the activity of the polymerase. The enzymatic activity of *Pfu* DNA polymerase was estimated based on the band intensity matching of the PCR amplicon to that of the commercial Thermo Scientific Fisher (TSF) and Promega (P) *Pfu* DNA Polymerase. One microliter of each diluted fractions as well as commercial polymerases were used in the PCR. The relative intensity of the PCR band as measured using ImageJ produced using 1 μL of the 5× diluted in-house *Pfu* DNA polymerase was estimated to be similar to that of the 2.5U Thermo Scientific *Pfu* Polymerase (TSF) (Fig. 3). Thus, the enzymatic activity for the current batch of *Pfu* DNA polymerase was estimated at 12.5 U/μL.

**Fig. 4.**
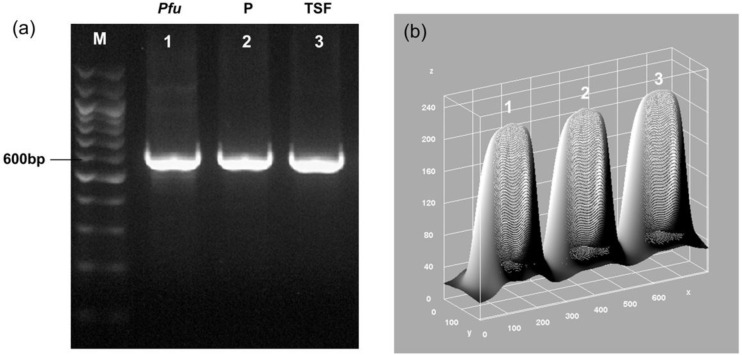
Analysis of *Pfu* DNA polymerase performance compared with commercial polymerases. (a) Gel electrophoresis of the PCR product to assess the functionality of the purified *Pfu* DNA polymerase in 1U. PCR amplification was carried out using p600 plasmid as the template and 1U of the purified *Pfu* DNA Polymerase, Promega *Pfu* DNA Polymerase and Thermo Scientific *Pfu* DNA Polymerase, respectively. Lane M: DNA size marker, Lane 1: 1U Purified *Pfu* Polymerase, Lane 2: 1U Promega *Pfu* Polymerase, Lane 3: 1U Thermo Scientific *Pfu* Polymerase. (b) 3D Surface Plot analysis on the intensity profile of the bands (axis units in pixels).

Furthermore, we also tested the activity of 1U from our 5× diluted *Pfu* polymerase with 1U of both commercial polymerases. We found the band intensity of the PCR amplicon using the purified *Pfu* DNA polymerase was similar to the commercial polymerases, Promega and Thermo Scientific Fisher (Fig. 4). The 3D surface plot analysis corroborated the analysis, which showed that the peaks and areas to be similar for all three bands. Collectively, an estimated 50000 units of *Pfu* DNA polymerase was obtained from a 400 ml of bacterial starter culture.

## DISCUSSION

The PCR method has changed the molecular biology field in a multitude of applications such as sequencing and diagnostics. In a DNA amplification process, one pivotal point that must be thoroughly considered is the accuracy of the copied DNA sequences. Common DNA polymerases have errors in their activity, limiting their applications in high fidelity PCR such as in amplification of long sequences. In applications DNA like sequencing, polymerases with proof-reading activities such as the *Pfu* is often the main choice (5). Subsequently, various modified polymerases were developed with a prime focus on *Pfu* to cater for a faster polymerization rate using fusions such as DNA binding proteins that essentially highlights the superiority of this proof reading enzyme (13). Alternatively, researchers have also opted for a combinational usage of *Pfu* and *Taq* DNA polymerase for high fidelity PCR with a high polymerization rate (2).

In this study, we cloned the *Pfu* DNA polymerase gene into the pSE420 plasmid. After successfully cloning the gene, we proceeded to overexpress the native *Pfu* DNA polymerase with standard IPTG induction protocol in the *E. coli* BL21 (DE3) pLysS for a tighter expression regulation as toxicity was reported in *Pfu* DNA polymerase overexpression on the *E. coli* BL21 (DE3) strain even when IPTG induction was not performed (11). Our study demonstrated an induction time of 5 hours for optimal protein expression. Previous publications using the standard shake-flask protein expression have collectively denoted *Pfu* polymerase induction time no longer than 5 hrs under IPTG concentration of 0.5–1 mM (7, 10, 14). We heated the lysate to denature and precipitate most of the host proteins. SDS PAGE analysis showed that the dialyzed soluble fraction lysate contains predominantly *Pfu* polymerase, which is retained due to its high thermostability while most of the bacterial proteins are denatured. Similar to our findings, other investigators have shown that the polymerase activity is not affected by host proteins (7, 15). Additionally, the removal of possible *E. coli* DNA contaminants can be applied depending on the research application by the use of Benzonase or DNAse treatment (16).

The functionality of the protein was assessed by standard PCR amplification using a plasmid as template for amplifying a 600 bp sequence. Dialysis of the soluble fraction which contains the in-house overexpressed *Pfu* DNA polymerase, removes small molecules and exchanges the buffer composition with that of the stabilizing buffer. The enzymatic activity of the purified *Pfu* DNA polymerase activity was estimated to be at 12.5U as determined by comparison with the known activity of the commercial *Pfu* DNA polymerase. This was carried out by diluting the protein to the common working concentration of 1U/μL and compared with the same concentration of the commercial *Pfu* DNA polymerases. Collectively, we have accumulated *Pfu* DNA polymerase with a total enzymatic activity of 50000 units from this method.

## CONCLUSION

The *Pfu* DNA polymerase was successfully overexpressed and purified with a simple heating step with minimal resources. We showed that the *Pfu* DNA polymerase is functioning and an estimated 50000 units of enzymatic activity was obtained from a 400 ml culture in our experimental setting. The purified *Pfu* DNA polymerase could be applicable for cloning and DNA sequencing applications.
